# Autoimmune hepatitis under the COVID-19 veil: an analysis of the nature of potential associations

**DOI:** 10.3389/fimmu.2025.1510770

**Published:** 2025-01-31

**Authors:** Chaojie Yu, Wenrui Wang, Qian Zhang, Zhenjing Jin

**Affiliations:** Department of Hepatopancreatobiliary Medicine, The Second Hospital of Jilin University, Changchun, Jilin, China

**Keywords:** SARS-CoV-2, COVID-19, autoimmune hepatitis, COVID-19 vaccine, immune-mediated liver injury

## Abstract

In recent years, the novel coronavirus infectious disease 2019 (COVID-19), caused by severe acute respiratory syndrome coronavirus-2 (SARS-CoV-2), has led to over 670 million infections and nearly 7 million deaths worldwide. The global pandemic of COVID-19 has precipitated a significant public health crisis. The prevalence of liver function abnormalities associated with SARS-CoV-2 is as high as 53% among healthy individuals or patients with autoimmune hepatitis (AIH) and shows a positive correlation with disease severity; moreover, specific adaptive immune responses can influence the trajectory and outcomes of COVID-19. For instance, SARS-CoV-2 may impact autoimmunity through mechanisms such as excessive stimulation of immune responses and molecular mimicry, particularly in genetically predisposed individuals. Currently, the overall mutational trend of SARS-CoV-2 indicates heightened infectivity and immune evasion capabilities. Consequently, vaccination remains crucial for universal protection against this disease. Nevertheless, alongside the widespread implementation of vaccination programs globally, an increasing number of cases have been documented where COVID-19 vaccination appears to trigger new-onset autoimmune hepatitis; yet definitive evidence is still pending elucidation regarding causality. In this review, we analyse the clinical-immunological characteristics, risks associated with severe disease progression, and prognosis for AIH patients infected with SARS-CoV-2; discuss the detrimental effects exerted by SARS-CoV-2 on hepatic function; summarise the mechanisms and attributes leading to new-onset AIH; as well as provide insights into how vaccination may interfere with autoimmunity processes. We continue to underscore the significance of vaccination while aiming to enhance awareness concerning potential risks associated with it—this could facilitate better management strategies for autoimmune diseases along with appropriate adjustments in vaccination protocols. Although the precise triggering mechanism linking COVID-19-related events to AIH remains unclear, existing evidence suggests that this relationship is far from coincidental.

## Highlights

- Clinical features, immunological characteristics, and risk assessment of SARS-CoV-2 infection in patients with autoimmune hepatitis (AIH) compared to healthy individuals.
- The detrimental impact of SARS-CoV-2 on hepatic function.
- Clinical features and possible mechanisms of new-onset AIH due to SARS-CoV-2.
- Evidence and mechanisms underlying AIH triggered by COVID-19 vaccination.

## 1 Introduction

Autoimmune hepatitis (AIH) is an immune-mediated chronic inflammatory liver disease with an increasing incidence worldwide. Its diagnosis is mostly based on serum biochemistry and liver biopsy. Currently recommended first-line therapies are immunosuppressive agents including corticosteroids and azathioprine. The mortality rate of COVID-19 is as high as 2-6%, which can be increased to some extent by advanced age, immunosuppressive status, individual susceptibility, and comorbidities with underlying medical conditions, including coronary artery disease, hypertension, diabetes mellitus, and chronic liver and renal disease.

Studies have shown that SARS-CoV-2 infection leads to abnormal levels of liver injury markers through mechanisms such as direct hepatocytopathy, severe inflammatory response, hypoxic or ischaemic injury complicated by endothelial lesions. This may promote the progression of the tendency of AIH to decompensation. It is thus clear that, especially in genetically susceptible populations, SARS-CoV-2 acts as a key trigger of autoimmunity, which can lead to autoimmune diseases through mechanisms such as immune overstimulation or molecular mimicry. Given the unavoidable immunosuppressive treatments and individual susceptibility, patients with autoimmune diseases have a higher prevalence of concomitant COVID-19 and a less conclusive prognosis. Meanwhile, SARS-CoV-2 infection may be one of the potential triggers of abnormal autoimmune activation (1).

Currently, WHO reports more than 170 vaccines, including protein subunits (PS), RNA, DNA, inactivated virus (IV), viral vectors (non-replicating) (VVnr), virus-like particles (VLPs), and viral vectors (replicating) (VVr), etc. Moreover, breakthroughs in the route of administration of vaccines have opened up new possibilities for specific immunity. For example, intranasal vaccines can trigger protective mucosal immunity (2). Yet, various new variants of SARS-CoV-2 make vaccine development a formidable challenge (3–5), such as Delta (B.1.617.2) and Omicron (B.1.1.529). Nonetheless, case reports of AIH induced after COVID-19 vaccination suggest a risk correlation between the two. Although the causal relationship is not clear, various reports suggest that the association may be more than coincidental.

Incidents of COVID-19 and hepatic autoimmunity have been reported, but different clinical features suggest complex and specific triggering mechanisms. In this review, we summarise the phenomenon of the association between COVID-19 and AIH, discuss the course and risk of SARS-CoV-2 infection in patients with AIH, and analyse the effects of SARS-CoV-2 and COVID -19 vaccination effects on hepatic autoimmunity and new onset of AIH. This provides new ideas for further research on basic mechanisms at the molecular immune level and control of vaccination with promising references.

## 2 Risk of SARS-CoV-2 infection in AIH patients

Autoimmune hepatitis is a dynamic, heterogeneous chronic immunoinflammatory liver disease (6). It can be divided into type I (60%-80%) and type II based on serum autoantibody profile. Clinically, they include chronic insidious, acute exacerbation and AIH-like liver injury diseases. Acute exacerbations often lead to diagnostic and therapeutic barriers due to the lack of typical manifestations, whereas AIH-like disease mostly lacks specific biological or histological manifestations.

Theoretically, patients with AIH may be at increased risk of SARS-CoV-2 infection and have a worse prognosis due to their genetic susceptibility and maintenance immunosuppressive therapy. In particular, patients with end-stage liver disease have an elevated rate of SARS-CoV-2 infection and mortality, as well as they are more susceptible to secondary infections, gastrointestinal bleeding, and other dangerous events. Whereas, the actual clinical impact of SARS-CoV-2 infection in patients with AIH is unclear. The available studies could not replicate the theoretical higher probability of SARS-CoV-2 infection in AIH patients, meaning that AIH patients are not at high risk of developing COVID-19, which contradicts the theoretical hypothesis.

For example, during the SARS - CoV - 2 epidemic in Northern Italy (7), about 26% of 136 AIH or AIH/PBC were suspected to be infected, 25% of patients had close contact with a suspected or confirmed case of COVID-19, and only 3% were confirmed cases, 96% of infected patients presented only no or mild-to-moderate respiratory symptoms. Even in the only fatal case, it was difficult to exclude interference from the underlying disease. The incidence of cases observed in this cohort was not significantly different from the estimated incidence in the general population and the prognosis was favourable. This suggests that patients with AIH are no more susceptible to COVID-19 than the general population and do not require reduction or cancellation of immunosuppressive therapy. The EUSurvey platform studied 1,779 patients with AIH across Europe (8). Of these participants, 2.2% (39/1779) were diagnosed with COVID-19, and the study ensured that there were no differences between infected and healthy individuals in terms of age, gender, AIH, cirrhosis status or post-liver transplantation status. The results showed that only one person developed severe pneumonia, indicating that the prevalence of COVID-19 is low and comparable to that of the general population. These results suggest that patients with AILD are not at high risk of developing COVID-19. In addition, Verhelst X et al. (9) followed up 85 patients with AIH in Belgium. 7 were suspected cases of COVID-19 and 1 was a confirmed case after liver transplantation and recovered well after non-invasive oxygen support. In conclusion, it is recommended that patients with AIH should pay full attention to prophylactic measures without discontinuing immunosuppressive therapy.

## 3 Effect of AIH on COVID-19

Theoretically, suppression of immunity could exacerbate the infection. Pre-existing abnormal autoantibodies could be one of the reasons for developing severe COVID-19. As Alessio Gerussi et al. (10) in Italy speculated that chronic immunosuppression may increase the incidence of severe COVID-19. A multicentre retrospective study included 254 patients with AIH (11). The study found that the application of systemic glucocorticoids (aOR 4.73, 95% CI 1.12-25.89) and thiopurines (aOR 4.78, 95% CI 1.33-23.50) as well as mycophenolate mofetil (aOR 3.56, 95% CI 0.76-20.56) and tacrolimus (aOR 4.09, 95% CI 0.69- 27.00) were all associated with COVID-19 progression.

Interestingly, a statistic combining data from three large registries (12) showed that immunosuppressive therapy in patients with AIH did not increase the risk of progression of COVID-19 to severe disease or death compared with other forms of liver disease and healthy patients. Similarly, Di Giorgio A (7) and Zecher BF et al. (8) conducted epidemiological investigations of COVID-19 at an early stage. They found that patients with autoimmune hepatitis, inflammatory bowel disease and cirrhosis were less severe or consistent with the normal population. This suggests that patients with AIH are not more susceptible to developing infections than the general population, demonstrating that the SARS-CoV-2 epidemic may not affect the course of immunotherapy in patients with AIH. In conclusion, there is no absolute correlation between immunosuppression and severe pneumonia. Even, D’Antiga L (13) indicated that the application of immunosuppressive agents had a protective effect on COVID-19 patients. Efe C et al. (14) found by retrospective analysis that the incidence of severe COVID-19 (15.5% vs. 20.2%, P = 0.231) and the all-cause mortality rate (10% vs. 11.5%, P = 0.852) were not significantly different between AIH and non-AIH patients. This result precisely indicates that maintaining immunosuppression during COVID-19 disease does not increase the risk of severe COVID-19 and reduces the risk of new liver injury during infection.

Currently, the main treatments for AIH are immunosuppressive agents such as azathioprine, corticosteroids, antimetabolites and liver transplantation (15). The concern is that hasty discontinuation and dose reduction may cause recurrence and exacerbation of AIH, leading to an increase in the dose of re-medication, while immunocompromise accordingly increases the rate of viral infection and the incidence of severe disease (16). In contrast, there are still more views on the adjustment of immunosuppression before and after COVID-19 disease course. Multiple case reports have shown that immunosuppression does not lead to worsening of COVID-19, so early dose reduction or replacement is still not recommended. The application of the lowest dose of maintenance therapy during infection is still the more dominant treatment regimen.

For example, Verhelst X et al. (9) investigated and followed up 110 AIH patients who were being treated during the COVID-19 epidemic. Finally, they recommended that AIH patients should pay full attention to prophylaxis without stopping immunosuppressive therapy. EASL and ESCMID published the opinion (17) that immunosuppressive therapy should not be altered in AIH patients before and after SARS-CoV-2 infection, as well as the strategy of reducing or changing immunosuppressive medications in advance during COVID-19 may lead to a poor prognosis (18). Not coincidentally, Lleo A et al. (19) questioned the argument that immunosuppressive drugs cause severe respiratory viral infections as shown in older data and concluded that empirical reduction of immunosuppressive agents is not justified. More precisely, the National Institutes of Health (NIH) (20) stated that oral steroid hormone therapy should not be discontinued before the diagnosis of COVID-19 is confirmed and that steroid hormone therapy should not be applied systemically after the diagnosis. What’s more, steroid hormone replacement therapy is available for patients with autoimmune liver disease combined with severe COVID-19.

This shows that immunosuppressive therapy for AIH may have an undeniable palliative effect on COVID-19. Nonetheless, the criteria for its application are still debatable. Small sample size, lack of a control group and short follow-up time are also factors that currently affect the reliability of the study in a precarious way.

It cannot be ignored that cirrhosis is an independent predictor of severe COVID-19 in patients with autoimmune hepatitis (P < 0.001; or, 17.46; 95% CI, 4.22-72.13) (14). Adjustment of immunosuppressive therapy during COVID-19 should adequately take into account the risk of derangement in high-risk patients. In the face of various conflicting and ambiguous views, perhaps an in-depth study of the underlying molecular biological mechanisms of the specific effect pathways of different immunosuppressive agents on the action of COVID-19 might be beneficial to our interpretation and understanding of this clinical epiphenomenon.

## 4 Triggering of AIH by SARS-CoV-2

### 4.1 SARS-CoV-2 disrupts innate immunity

**Figure 1:**
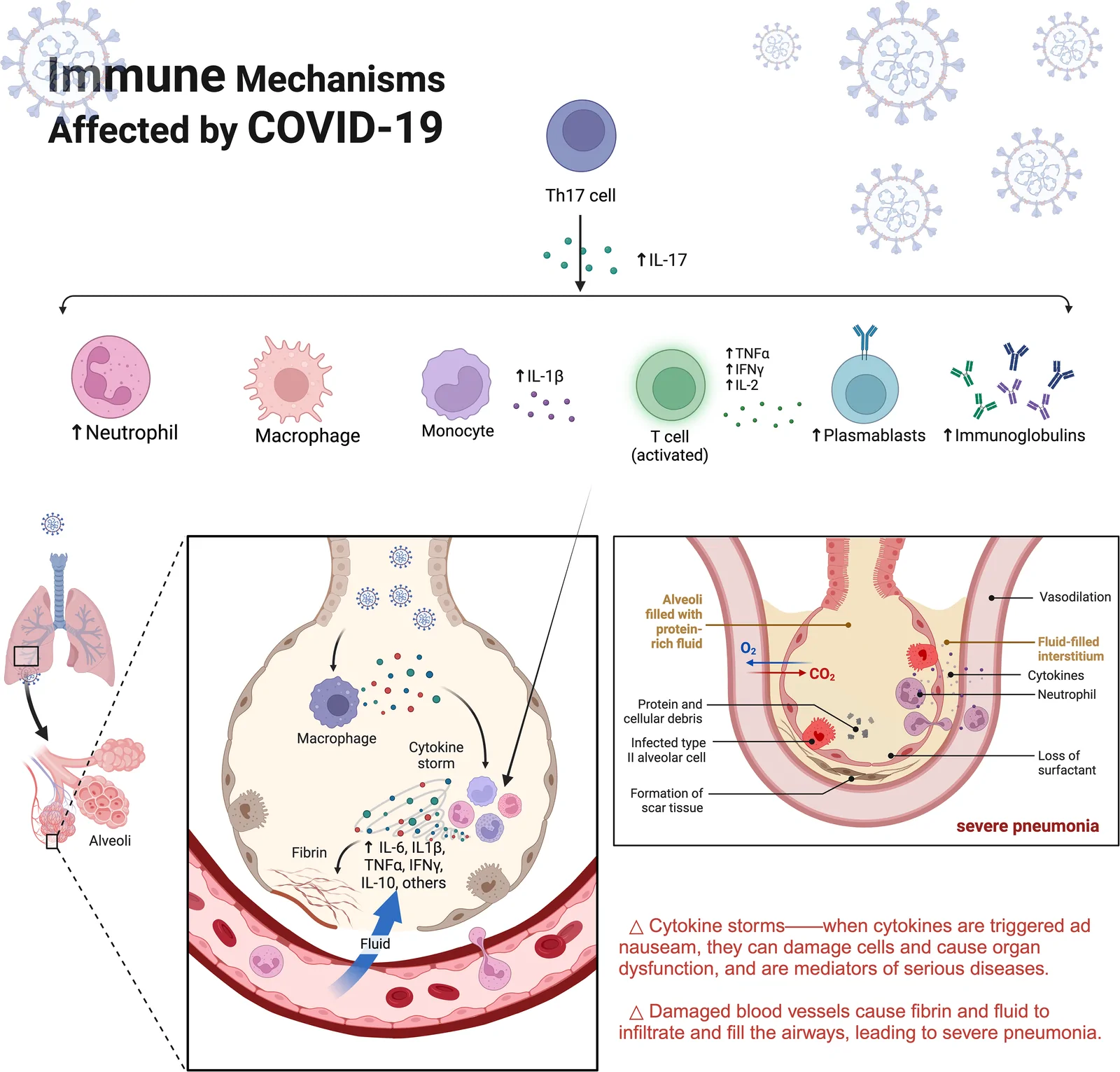
Immune Mechanisms Affected by COVID-19. SARS-CoV-2 infects lung tissue through the respiratory tract and over-excite the immune response. Recruitment of immune cells such as aggregated macrophages recognises the virus and produces large quantities of cytokines, creating an inflammatory cycle that results in destruction of lung tissue. IL-6, Interleukin-6;IL1-β,Interleukin-1β;TNF-α,tumour necrosis factor-α;IFNγ, Interferon γ.

SARS-CoV-2 infection can disrupt innate antiviral immune defences, leading to a massive release of cytokines (21–24). Severe cases show excessive elevation of blood concentrations of pro-inflammatory factors and chemokines such as IL-6, MCP1, TNF-α and IFN-γ. Over-activation of immunity leads to systemic cascade reactions such as acute respiratory distress syndrome, septic shock, multi-organ failure and even death (25–27).Using mathematical modelling, S.Q.Du (28) et al. demonstrated that delaying the onset of adaptive immunity in the early stages of the infection may be a promising therapeutic tool for patients at high risk of COVID-19 (**Figure 1**).

### 4.2 SARS-CoV-2 interferes with autoimmunity

Viruses can induce changes in the clinical features and progression of deterioration in a variety of autoimmune diseases. For example, herpesvirus-induced systemic lupus erythematosus (SLE), multiple sclerosis (MS), rheumatoid arthritis (RA), and hepatitis C virus-associated cryoglobulinaemic vasculitis (29, 30). This implies that viruses have some autoimmunomodulatory role, such as coxsackievirus B, rotavirus (31), influenza A virus as well as measles, rubella, and EBV (32). Similarly, SARS-CoV-2, a novel enveloped single-stranded RNA β-coronavirus, has been reported to induce diseases such as Guillain-Barré syndrome (GBS) (33), autoimmune haemolytic anaemia, anti-MDA-5 positive dermatomyositis (34), arthritis, psoriasis (35), and immune thrombocytopenic purpura (36).

### 4.3 Polyclonal B cell activation

COVID-19-mediated autoimmunity may be plausible. According to type II hypersensitivity, the production of autoantibodies after viral infection may lead to tissue damage (cross-reactivity). This is a possible mechanism of virus-induced autoimmunity. But it is not certain whether COVID-19-associated slave autoantibodies may act as a catalyst for disease exacerbation, or a mediator for triggering immune-disordered inflammation, or simply a concomitant phenomenon of intense inflammation. Studies have shown (37) that 10%-15% of COVID-19 patients have circulating autoantibodies, such as anti-type I interferon autoantibodies, antinuclear antibodies, lupus anticoagulant, anti-SSA/Ro antibodies (38), anticardiolipin antibodies (39), anti-IFN antibodies (40), dermatomyositis-associated anti-MDA5 antibodies, and anti-angiotensin converting enzyme 2 (ACE2) autoantibodies. As the disease worsens an imbalance occurs which in turn damages systemic tissues (41, 42). This phenomenon suggests that antibody activation of COVID-19 may be one of the factors triggering AIH. It is important to note that autoimmune dysregulation is more likely to occur in the elderly or in people with comorbid underlying diseases. Defective T-cell activation due to aging of the organism’s microenvironment is hardly implicated (43).

Interestingly, there is no direct evidence that SARS-CoV-2 can directly cause AIH. Bartoli A et al. (41) published a report that middle-aged women with Hashimoto’s thyroiditis developed primary sclerosing cholangitis and Guillain-Barré syndrome after infection with SARS-CoV-2. Specific manifestations were elevated liver enzymes and a positive AMA (1:640) as well as a liver biopsy showing Ludwig stage I, which is consistent with early manifestations of PBC. Certain patients may develop sero-immunological features of AIH during the course of COVID-19. Exceptionally, IgG elevation in AIH after SARS-CoV-2 infection was not significant. Whereas, given that Hartl et al. (44) reported a 10% probability of the presence of a subgroup of IgG-normal AIH, normal IgG is not a major basis for ruling out AIH.

### 4.4 SARS-CoV-2 causes liver injury

What has been identified, but not yet definitively reported, is the presence of hypoalbuminaemia, elevated gamma-glutamyltransferase and alkaline phosphatase during the course of COVID-19 patients. Of these, 2-11% of COVID-19 patients had hepatic comorbidities, and 14-53% had abnormal levels of liver injury markers detected during the course of the disease (45). EASL-ESCMID (46) suggested that patients with severe pneumonia were more likely to have abnormal transaminases. Despite all this, transaminase abnormalities have not been shown to be a causative factor for COVID-19 exacerbations. Possible mechanisms include (45) immune-mediated cytokine storm, pneumonia-associated ischaemic-hypoxic injury, microthrombosis, and drug effects such as lopinavir (47). Interestingly, the direct cytotoxic effects of the virus are negligible because the viral titre in liver tissue is relatively low and viral inclusion bodies have not been detected. In addition, the genome sequence homology of the viruses seems to validate this phenomenon. For instance, 60% of SARS (85% homozygosity) can lead to hepatic impairment (48) and MERS-CoV (50% homozygosity) infection carries a risk of causing lobular hepatitis (49).

**Figure 2:**
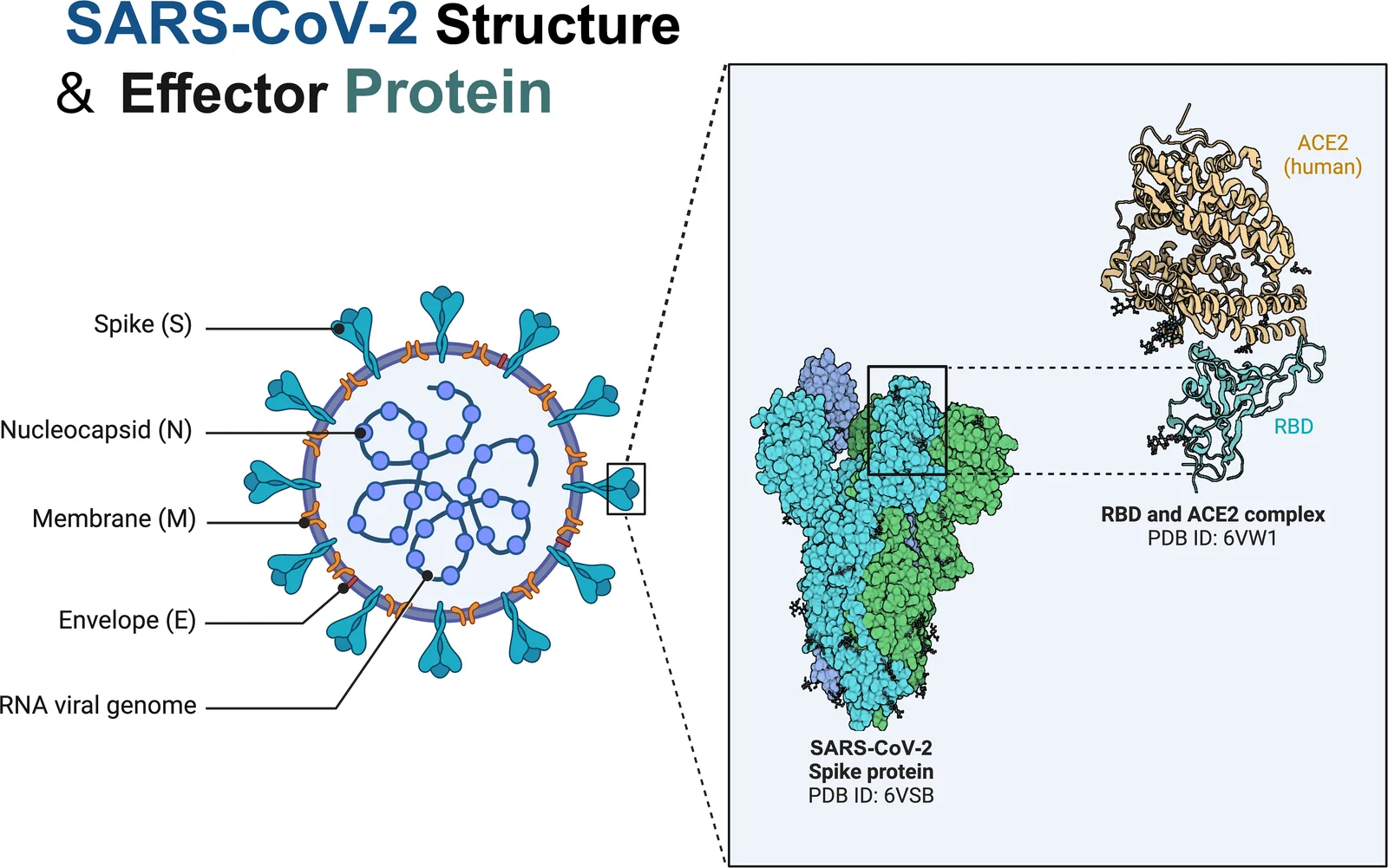
Structure and Spike protein of SARS-CoV-2. SARS-CoV-2 is a novel enveloped single-stranded RNA beta coronavirus named by its characteristic crown-like structure under electron microscopy, which belongs in the family Coronaviridae and can cause disease in mammals and birds with a crown-like structure created by surface spike proteins. Coronavirus spike (S) protein mediates viral entry into cells by binding to cellular receptors and mediating membrane fusion. RBD, receptor-binding domain; SARS-CoV-2, severe acute respiratory syndrome coronavirus 2.

**Figure 3:**
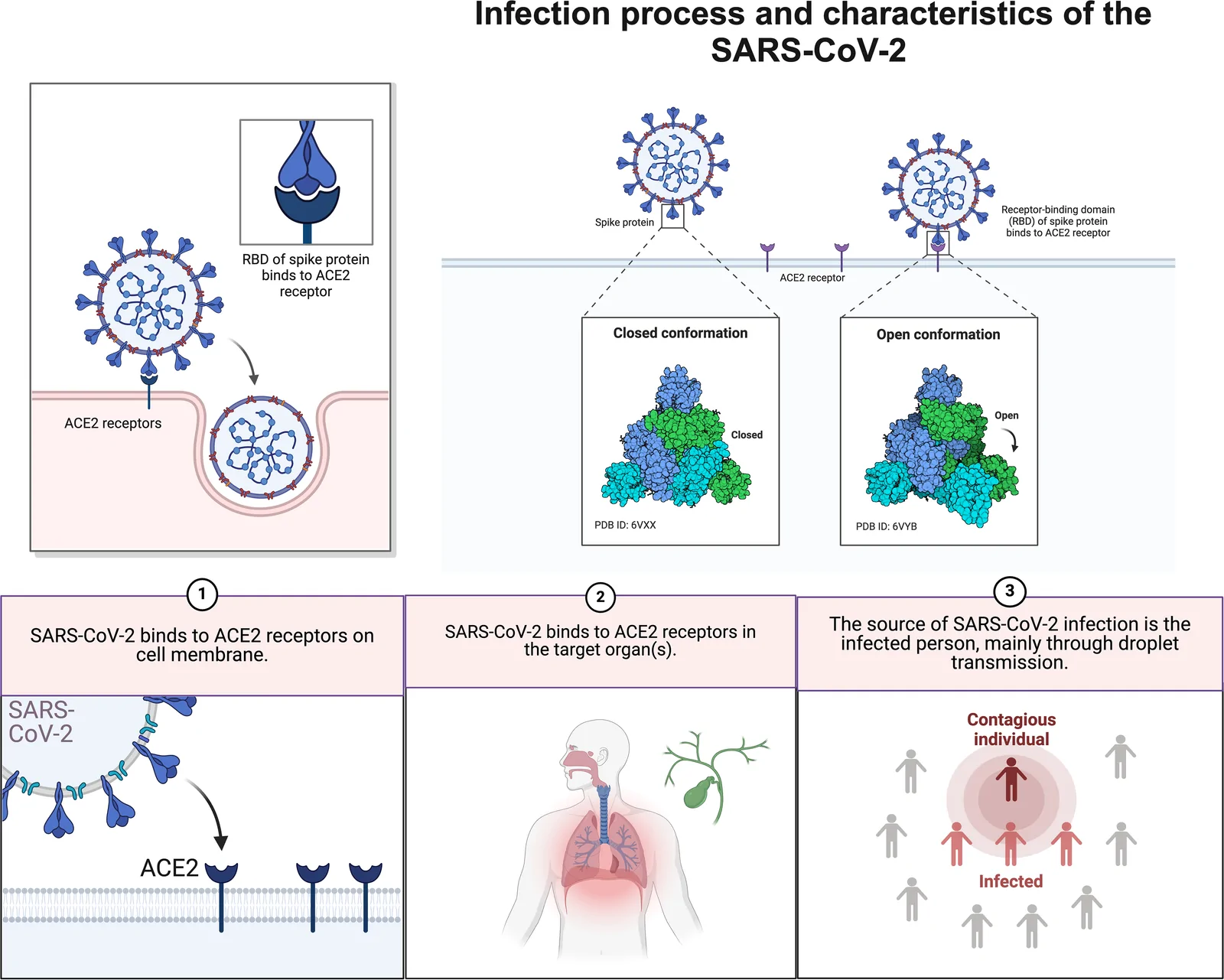
Infection process and characteristics of the SARS-CoV-2. SARS-CoV-2 binds to ACE2 receptors on host cells in the respiratory tract and spreads when an infected person sheds the virus.ACE2,Angiotensin-converting enzyme 2.

Gamma-glutamyltransferase is known to be a diagnostic biomarker of bile duct cell injury. Coincidentally, cholangiocytes show high expression of ACE2 receptor (50) and the spike (S) protein of SARS-CoV-2 binds to angiotensin converting enzyme 2 (ACE2) receptor (51). Subsequently, transmembrane serine protease 2 catabolism of the S protein occurs after the binding body enters the target cell (**Figure 2**). Following this, the viral genome is released from the endosomes and later replicates and proliferates (**Figure 3**). Notably, viral RNA has only been detected in faecal and blood samples, which is not sufficient to fully justify this mechanism. Therefore, further monitoring of the effect on the degree of cholestasis and biliary tract damage is still required.

### 4.5 Homology of peptide sequences

Homologous self-proteins of virally shared peptide sequences can lead to pathological autoimmune responses. Based on this principle, Kanduc D et al. explored the possibility of molecular mimicry induced autoimmune diseases by quantitative analysis of peptide sharing (52). Coincidentally, Vorjani et al. (53) pointed out through an *in vitro* study that molecular mimicry is the cross-reactivity of antibodies against SARS-CoV-2 spike glycoprotein S1 with structurally similar host peptide protein sequences. The peptide sequences included the high-affinity human histone transglutaminase, nuclear antigen, anti-extraction nuclear antigen and myelin basic protein. This suggests that molecular mimicry is involved in the SARS-CoV-2-induced autoimmune response.

**Figure 4:**
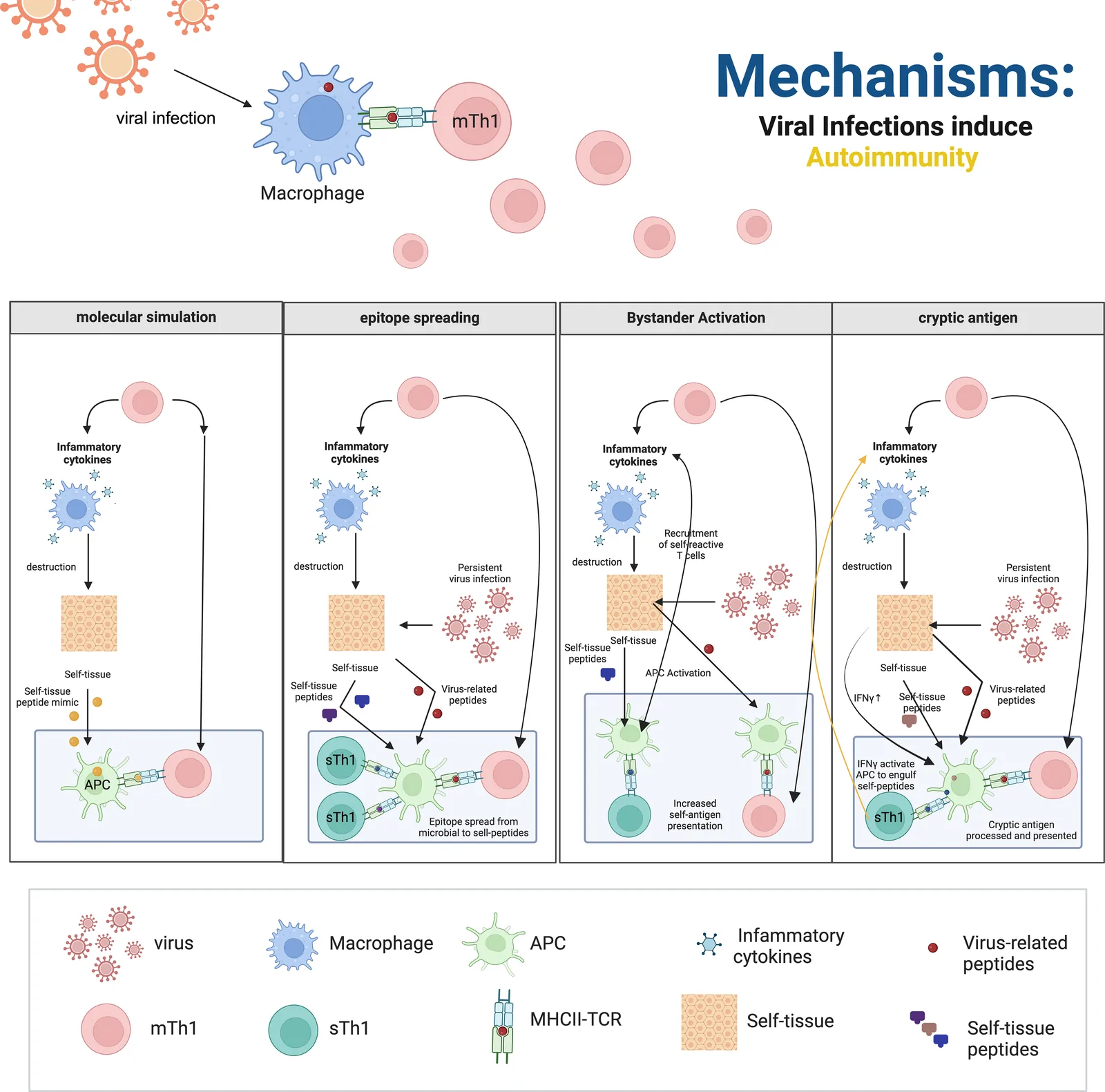
Mechanisms of autoimmunity induced by viral infection. Activated specific Th1 (mTh1) cells migrate to the infected organ following viral infection. Molecular mimicry refers to the induction of an immune response by viral infection including activation of mTh1 and cross-reactive Th1 cells, cause the release of inflammatory factors and recruit macrophages, which mediate self-tissue damage, then self-tissue antigens release with processed by APCs, Leads to cross-reactivity of homologous autologous proteins with viral shared peptide sequences, leading to a sustained autoimmune response. Epitope spreading refers to the activation of mTh1 cells with persistent viral infection, self-antigens release after self-tissue injury which are taken up by APCs and presented to sTh1 cells, and result persistence of an auto-reactive immune response that spreads to multiple sTh1s. Bystander activation refers to the non-specific activation of sTh1 cells, where the activation of mTh1 cells leads to an increase in the infiltration of T-cells and the activation of sTh1 cells by T-cells, leading to a sustained auto-immune response. Cryptic antigen refers to the secretion of IFN-γ by mTh1 cells, which activates and promotes phagocytosis of self-antigens by APCs and promotes the production of proteases that differentially process self-antigens for presentation (cryptic epitopes activate sTh1 cells and mediate the destruction of their own tissues). APCs, antigen-presenting cells; MHC II, major histocompatibility complex class II; TCR, T-cell receptor.

In addition, the same immune response may be triggered between COVID-19 and AIH by breaking immune tolerance (54). It is now known that SARS-CoV-2 can upregulate autoimmunity through molecular mimicry, epitope spreading, bystander activation (32), immortalisation of infected B cells, cryptic antigens, spiny synapse-mediated super antigenic activity, and inflammatory signalling stimulation (**Figure 4**). Note that the efficacy of these effects depends on a variety of factors such as genetic background such as congenital B-cell autoimmune defects, viral strain type, viral load, host immune status and duration of infection.

### 4.6 Gut-lung axis

Microbiomic analysis of COVID-19 suggested the presence of a gut-lung axis as well as autoimmune-associated microbial alterations. Nagata N et al. (55) found the presence of a gut-lung axis in COVID-19 by multi-omics analysis, demonstrating the existence of multiple gut microbial-metabolite-cytokine interrelationships. Katz-Agranov N et al. (56) found a loss of biodiversity, an increase in the number of pathogenic bacteria (e.g., Aspergillus), and a relative decrease in the number of commensal bacteria (anti-inflammatory, protective microorganisms, e.g., Lactobacillus) in both by comparing the microbiomes of COVID-19 and lupus. Analyses of the microbiomes of COVID-19 and AIH are still lacking. It’s worth noting that due to similar pathogenic mechanisms, the findings of the classic autoimmune disease lupus are informative about the triggers of AIH.

Genetic association studies can help identify genes and biological pathways behind specific physical outcomes or traits. AIH is known to be rich in genetic susceptibility. According to typical biomolecular mechanisms, the immune mechanisms such as risk of infection, disease progression, or cytokine triggering in SARS-CoV-2 must involve regulation and variation at the level of deep genetic pathways. Regrettably, there is a lack of cross-targeting results at this stage. In the future, improved genetic studies may further advance the exploration of the causal association between COVID-19 and autoimmune diseases such as AIH.

## 5 COVID-19 vaccination-related AIH

COVID-19 caused an international pandemic in a short period of time. Measures such as quarantine, lockdown and masking were taken by various sectors to combat the rapid spread of SARS-CoV-2. Considering the fundamental ways of epidemic prevention and the actual needs of the society, the development of vaccines is a promising way to block asymptomatic transmission (57). Vaccines are known to be important in infectious diseases such as influenza, measles and tetanus. Strong evidence also exists to demonstrate that vaccination with COVID-19 reduces the rate of infection and severe disease. People are still in the sceptical stage of vaccine development techniques and side effects. In particular, cases of vaccine-induced AIH have recently been reported (58), and the frequency of such adverse events has deepened the public’s scepticism towards the vaccine. The specific mechanism and causality of AIH induced by COVID-19 vaccine still need further study. Therefore, we summarise the characteristics of COVID-19 vaccine-induced AIH based on the current evidence and discuss the possible mechanisms.

### 5.1 Clinical features of AIH induced by COVID-19 vaccine

It has long been shown that hepatitis A, human papilloma and influenza virus vaccines (59) can induce AIH (60). The vaccine carries a greater risk of causing liver injury in people with allergies, a history of post-vaccination immune disorders, autoimmune disorders and a genetic family history (61). Vaccine development departments safeguard the safety of vaccines by conducting rigorous clinical drug trials. Nonetheless, as the vaccine became widely available, a series of confirmed cases of AIH were reported around the globe (**Table 1**).

**Table 1:** Characteristics of patients with autoimmune hepatitis after COVID-19 vaccination.

Reference	Age	Gender	Vaccine	Latency	Comorbidities	Concurrent medication	Treatment	Follow-up time	Mitigation
Nik Muhamad Affendi NA et al. (62)	63	F	AZD1222	14	Ulcerative Colitis (UC) and Primary Sclerosing Cholangitis (PSC)	Ursodeoxycholic acid, mesalazine, atorvastatin	Prednisolone (40 mg)	2 weeks	Yes
Fimiano F et al. (63)	63	F	BNT162b2 mRNA (Pfizer Biotech)	23	Postmenopausal hypothyroidism	--	Methylprednisolone	--	Yes
Palla P et al. (64)	40	F	Pfizer-BioNTech mRNA Vaccine	30	Sarcoidosis	--	Prednisolone (40 mg)	5 months	Yes
Bril F et al. (65)	35	F	Pfizer Biovaccines-first dose	7	Gestational hypertension, third trimester	Labetalol	Prednisolone	--	Yes
Lodato F et al. (66)	43	F	m-RNA-BNT162b1 (Pfeizer Biontech)-second dose	15	Mild dyslipidaemia	Ginkgo biloba	Methylprednisolone (1 mg/kg/day)	8 weeks	Yes
Shahrani S et al. (67)	72	F	Pfizer-BioNTech mRNA Vaccine-booster injection	10	N	--	--	--	--
Shahrani S et al. (67)	59	F	AZD1222	12	Dyslipidaemia	Supplements	--	--	--
Boettler T et al. (68)	52	M	BNT162b2mRNA vaccine-first dose	10	Hypothyroidism	Levothyroxine	Budesonide (9 mg), ursodeoxycholic acid	8 weeks	Yes
Pinazo-Bandera JM et al. (69)	77	F	Comirnaty vaccine-second dose	2	--	Bromazepam, losartan, and omeprazole	Prednisone (60 mg/day)	6 months	Dead
Pinazo-Bandera JM et al. (69)	23	M	Spikevax vaccine-second dose	10	--	--	Prednisone (60 mg/day)	3 months	Yes
Kang SH et al. (70)	27	F	Pfizer Biovaccines-second dose	7	--	--	Prednisolone (40 mg/day)	--	Yes
Cao Z et al. (71)	57	F	CoronaVac-first dose	14	--		Methylprednisolone, azathioprine, ursodeoxycholic acid	5 months	Yes
Avci E et al. (72)	61	F	Pfizer/BioNTech BNT162b2 mRNA Vaccine	30	--	--	Prednisolone (40 mg), Azathioprine (AZA)	1 month	Yes
Garrido I et al. (73)	65	F	Moderna-COVID-19-first dose	14	JAK2 V617F-positive polycythemia vera	Polyethylene glycol interferon, acetylsalicylic acid, sertraline, esomeprazole	Prednisolone (60 mg)	1 month	Yes
Zhou T et al. (74)	36	F	Moderna mRNA-1273-first dose	11	PSC, ulcerative colitis	Ursodeoxycholic acid	Prednisone (50 mg), azathioprine (75 mg)	2 weeks	Yes
Ghielmetti M et al. (75)	63	M	mRNA-1273-first dose	7	Type 2 diabetes and ischaemic heart disease	Metformin, acetylsalicylic acid, lovastatin	Prednisone (40 mg)	2 weeks	Yes
McShane C et al. (76)	71	F	Moderna mRNA vaccine-first dose	4	Cholecystectomy, left total hip replacement, osteoarthritis, knee osteoarthritis	Paracetamol (within 24 hours of vaccination)	Steroids	--	Yes
Clayton-Chubb D et al. (77)	36	M	ChAdOx1 nCoV-19 vaccine (Oxford-AstraZeneca)-first dose	26	Hypertensive disease, post laser eye surgery.	Olmesartan, fluoroquinolone eye drops, acetaminophen, ibuprofen	Prednisolone (60 mg)	1 month	Yes
Tan CK et al. (78)	56	F	ModernaCOVID-19 vaccine (mRNA-1273)-first dose	1	--	Lovastatin	Budesonide	--	Yes
Londoño MC et al. (58)	41	F	Moderna vaccine (mRNA-1273)-first dose	1	premature ovarian failure	Hormones	Prednisone (1 mg/kg)	--	Yes
Rocco A et al. (79)	80	F	Pfizer-BioNotex Vaccine	14	Hashimoto's thyroiditis	Levothyroxine, pravastatin, aspirin	Prednisone (1 mg/kg/day)	--	Yes
Zin Tun GS et al. (80)	47	M	Moderna vaccine-first dose	3	N	N	Prednisolone (40 mg/day)	2 weeks	Yes
Lee SK et al. (81)	57	F	Pfizer/BioNTech BNT162b2 mRNA Vaccine-first dose	14	--	--	Ursodeoxycholic acid (13 mg/kg)	2 weeks	Yes

**Figure 5:**
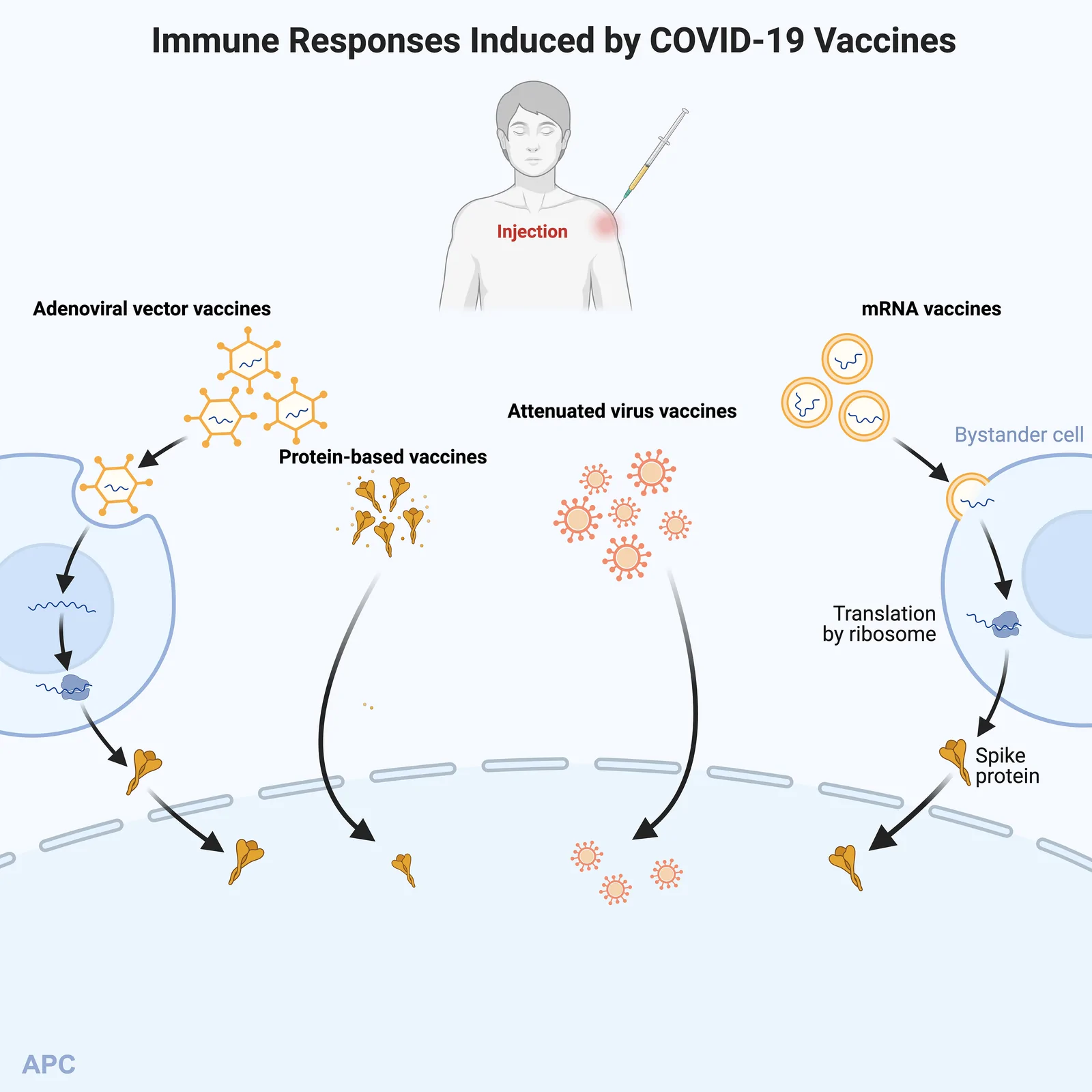
Immune Responses Induced by COVID-19 Vaccines. Different types of COVID-19 vaccines activate the body’s immune response in different ways. The mRNA vaccines (e.g., mRNA-1273) are delivered to bystander cells via lipid nanoparticles. mRNA encodes, transcribes, and translates modified spike proteins, which are secreted and then taken up and processed by the antigen. Adenoviral vector vaccines contain cDNAs encoding full-length spike proteins. cDNAs are transcribed into mRNAs in the nucleus and translated into spike proteins in the cytoplasm. Protein vaccines consist of the spike protein and adjuvant, and attenuated virus vaccines contain whole inactivated virus particles and adjuvant. The latter two can be directly processed by APC. Then APC activates the cellular response after presentation of the signal peptide. APC, antigen-presenting cell.

Fimiano and Tan CK et al. (63, 78) reported the types of COVID-19 vaccines that can induce AIH, including the second dose of AstraZeneca’s vaccine (AZD1222), Pfizer-BioNTech’s mRNA vaccine, Moderna’s COVID-19 vaccine (mRNA-1273), and CoronaVac’s vaccine followed by the third dose of Pfizer Biotech’s Technologies’ booster vaccine third dose. The main types of vaccines that can induce AIH in the cases reported so far include mRNA vaccines (most), viral vector vaccines and inactivated vaccines (least) (58, 65, 76, 77) (**Figure 5**).

Bril et al. (65) reported the first case of new onset of AIH after vaccination in a 35-year-old postpartum woman who developed generalised pruritus, hepatomegaly, bilirubinuria, jaundice, and antinuclear antibody positivity (1:1,280) one week after vaccination with the COVID-19 vaccine (Pfizer Biotech, first dose). Exceptionally, the patient did not have elevated IgG levels and a liver biopsy suggestive of eosinophilia (44). Therefore, we cannot exclude hepatotoxic effects of the drug.

Clayton-Chubb et al. reported the case of a 36-year-old male physician of Iraqi origin (77), who had hypertension but no liver disease. Twenty-six days after the patient received his first dose of ChAdOx1 nCoV-19 vaccine (Oxford-AstraZeneca), he developed abnormal liver function (ALT, AST, γ-glutamyltransferase, etc.). He was positive for antinuclear antibodies (titre 1:160). The biopsy showed interfacial hepatitis with mixed inflammatory cell infiltration, which was consistent with the presentation of AIH. Of note, the patient’s immunoglobulin G was also not elevated. Obvious confounding factors such as pregnancy and other underlying medical history were ruled out in this case. This supports the idea that the COVID-19 vaccine induces autoimmune phenomena and is not related to the mechanism of action of the vaccine. Undeniably, despite the patient’s good health literacy, we cannot exclude the role of confounding factors such as drugs or toxicants.

Recently, Ghielmetti M et al. (75) reported a case of a 63-year-old male with no history of autoimmunity or natural SARS-CoV-2 infection. He developed acute severe autoimmune liver disease 7 days after the first dose of mRNA-1273. The patient’s immunoglobulin G was mildly elevated. Exceptionally, the patient’s AMA differed from the conventional PBC pattern and also had a unique ANA. he carried the PBC protective HLA DRB1*11:01 allele (82), whereas the determinants of AIH susceptibility are mainly the HLA alleles DRB1*03 and DRB1*04. The clinical significance of these new specific results needs to be further evaluated.

Cases of liver injury due to Pfizer/BioNTech BNT162b2 mRNA COVID-19 vaccine have been reported in the UK. Not coincidentally, a case of drug-induced liver injury due to COVID-19 vaccine was reported by Mann R (83) et al. A 61-year-old woman developed jaundice and malaise 9 days after vaccination (Pfizer, second dose). Her laboratory parameters showed elevated alkaline phosphatase and bilirubin. Inconsistently, the patient’s antibodies were all negative, and a liver biopsy showed only mild oedema and scattered infiltration of inflammatory cells, which is not consistent with the inference that the vaccine causes autoimmune liver disease. Of course, this study does not rule out a correlation with the patients’ history of irritable bowel, cholecystectomy, and fatty liver.

There are many similar reports (**Table 2**). For example, Lee SK et al. reported a 57-year-old woman who was diagnosed with AIH-PBC overlap syndrome 2 weeks after vaccination (Pfizer/BioNTech BNT162b2 mRNA vaccine, first dose) (81, 84). In addition, Mekritthikrai et al. (85) reported a case of new-onset AIH in a 52-year-old woman after 2 doses of COVID-19 inactivated vaccine (CoronaVac). Although an increasing number of cases suggest an association between COVID-19 vaccine and the development of AIH, the mechanism remains unclear.

**Table 2:** Symptoms, laboratory tests, pathological findings in patients with autoimmune hepatitis after vaccination.

Reference	symptom	ALP(U/L)	AST/ ALT(U/L)	Total bilirubin (mg/dl)	IgG (mg/dL)	antibody	Liver biopsy findings
Nik Muhamad Affendi NA et al. (62)	Jaundice, itching	299	505/ 354	18.3	2030	ANA (1:320)	Potential PSC and bridging fibrosis, interface and lobular hepatitis
Fimiano F et al. (63)	Abdominal pain, nausea, hyperpigmented urine, jaundice, diarrhoea	273	1625/ 1778	18.6	1760	(-)	Severe acute hepatitis with plasma cell-rich inflammation, interfacial activity and focal fusion necrosis of the hilar/periportal and hepatic lobules
Palla P et al. (64)	Transaminasemia	——	——	4.8	240	ANA (1:640)	Active hepatitis with necrotising inflammation and severe lobular inflammatory infiltrate at the liver interface, periportal/periportal fibrosis with occasional bridging of fibrous septa
Bril F et al. (65)	Jaundice, generalised itching	170	754/ 2001	——	1081	ANA (1:1280),double-stranded DNA Antibody (1:80)	Consistent with AIH
Lodato F et al. (66)	Jaundice and itching	——	51/ 52	17.54	179	(-)	Moderate inflammatory infiltrate of the portal vein, interfacial hepatitis, biliary tract injury, mild ductal hyperplasia, hepatic lobular punctate necrosis.
Shahrani S et al. (67)	--	125	1452/ 2280	1.7	1940	AMA	Lobular inflammation and hepatocellular necrosis with lymphocytic and plasma cell infiltration of the portal vein
Shahrani S et al. (67)	Jaundice	189	962/ 1178	7.37	1740	(-)	Lympho-plasma-cellular portal vein infiltration without biliary features
Boettler T et al. (68)	Weakness, loss of appetite, itching of skin, jaundice	142	——/ 2130	7.7	——	ANA (1: 200),AMA-M2,SMA	Interfacial hepatitis with moderate lymphoplasmacytic infiltration and lobular necrosis and foci of apoptosis
Pinazo-Bandera JM et al. (69)	Vomiting, disorientation, scleral jaundice	159	474/ 552	3.1	N	ANA (1: 160),AMA-M2,HLA-DR4 (+)	--
Pinazo-Bandera JM et al. (69)	Mononucleosis syndrome-like symptoms, jaundice	202	702/ 587	2.3	1647	HLA-DR3 (+)	Consistent with AIH
Kang SH et al. (70)	Nausea, vomiting, headache, fever, jaundice	182	1004/ 1478	8.6	1641	ANA (1:80)	Lymphocyte and plasma cell clusters and a few eosinophils infiltrating the portal vein and lobules, diffuse moderate necroinflammatory injury of the hepatic lobules, periportal hepatocellular degeneration, mild cholestasis
Cao Z et al. (71)	Generalised itching, jaundice	212	819/ 974	16.6	1744	ANA (1:640),AMA,ASMA	Hepatic fibrosis (stage 2) and active hepatitis (grade 2) with moderate to severe interfacial necroinflammation, severe lobular lymphocytic/lymphoplasmacytic infiltration, hepatic stye formation
Avci E et al. (72)	--	436	913/ 455	11.8	4260	ANA (1: 100)、ASMA (1: 100)	Hepatic sinusoidal stenosis, severe lymphocytic infiltration in the hilar and periportal areas, periportal interface hepatitis, mild fibrosis
Garrido I et al. (73)	Abdominal pain, jaundice	24	1056/ 1092	1.14	↑	ANA (1: 100)	Inflammatory infiltrate resulting in marked dilatation of the portal vein and plasma cell aggregates
Zhou T et al. (74)	Mild muscle pain	↑	581/ 588	1.4	2475	ANA (1:2560),anti-ds DNA	Hepatoportal lymphocytic, plasma cell, and eosinophilic infiltrate, interfacial hepatitis, discrete florid formation, hepatocyte apoptosis, bile duct reaction
Ghielmetti M et al. (75)	Jaundice, malaise and loss of appetite	192	1127/ 1038	11.98	1996	ANA(1:640),APCA,anti-SARS-Cov2IgG spike 1 antibody,β2-GP1IgA	Inflammatory portal infiltrate, lobular and centrilobular inflammation, centrilobular necrosis
McShane C et al. (76)	Jaundice	237	1469/ 1143	15.79	2177	ASMA	Marked polymorphic dilatation of 20 hepatic hilar ducts, polymorphic inflammatory cell infiltrate (plasma cells, lymphocytes, eosinophils, neutrophils, PASD-positive residual macrophages), necrosis of portal-hepatic and centrilobular hepatic bridging
Clayton-Chubb D et al. (77)	N	118	633/ 1774	0.99	N	ANA (1:160)	Interfacial hepatitis with mixed inflammatory cell infiltrate (lymphocyte predominance)
Tan CK et al. (78)	Jaundice, malaise, persistent anorexia	298	1124/ 1701	5.96	3260	ANA、ASMA	Marked hepatic lobular inflammation, interfacial hepatitis, plasma cell aggregation, stromal formation, hepatocyte apoptosis, early fibrosis of the portal vein
Londoño MC et al. (58)	Epigastric pain, nausea, vomiting, jaundice	190	993/ 1312	2.3	2080	ANA (1:80)、ASMA、SLA	Marked dilatation of hepatic portal ducts, dense inflammatory infiltrate (lymphocytes and plasma cells), severe interfacial hepatitis and lobular inflammation, scattered necrotic cells, apoptotic bodies
Rocco A et al. (79)	Jaundice	243	1401/ 1186	10.5	3500	ANA (1:160)	Interfacial hepatitis, moderate lymphoplasmacytic infiltrate, multiple foci of confluent lobular necrosis, Quantam bodies
Zin Tun GS et al. (80)	Jaundice	229	——/ 1048	11.11	2510	——	Acute active hepatitis, extensive areas of bridging necrosis, marked interfacial hepatitis, lymphoplasmacytic inflammation with eosinophils, ballooning hepatocytes, multinucleated giant cells and pericytes
Lee SK et al. (81)	Fatigue and general malaise	250	635/ 870	1.2	1532	ANA (1:160),AMA-M2	Consistent with overlap syndrome, moderate hepatoportal inflammation, interfacial hepatitis, florid formation and sheet necrosis, non-suppurative and granulomatous biliary destruction and proliferation

ALP,alkaline phosphatase;AST,aspartate Transaminase;ALT,amino Alanine Transferase;IgG,Immunoglobulin G;ANA,antinuclear antibody;AMA,anti-mitochondrial antibody;SLA,Anti-soluble Liver Antigen Antibody;HLA-DR,Human leukocyte antigen DR allele;PSC,primary sclerosing cholangitis;AIH,autoimmune hepatitis.

### 5.2 Mechanisms of vaccine-induced AIH

Vaccines, as biological agents that render organisms immune to disease, are known to trigger regular or irregular autoimmune responses. The prevailing mechanisms (86) include molecular mimicry, bystander activation, central tolerance defects, adjuvants, epitope spreading and sustained antigenic stimulation.

#### 5.2.1 Molecular mimicry

Molecular mimicry is the significant homology of amino acid sequences between a vaccine determinant cluster and its own antigen. This property may lead to the synthesis of anti-spiking antibodies. Anti-spiking antibodies will cross-react immunologically with structurally similar host peptide proteins (86) thus leading to AIH. Both AdV and mRNA vaccines encode SARS-CoV-2 spike (S) proteins, the primary target of neutralising and therapeutic monoclonal antibodies produced by natural infection (87, 88). kanduc et al. proposed that the human body has a SARS-CoV-2 spike glycoprotein that share the heptapeptide (52). Certainly, there have been cases reported that a variety of vaccines can trigger different autoimmune diseases through molecular mimicry, e.g., human papillomavirus (HPV) triggers systemic lupus erythematosus, influenza (H1N1) vaccine leads to Guillain-Barre Syndrome (GBS), and Hepatitis B virus vaccine leads to multiple sclerosis (MS) (86).

Studies have shown that infectious diseases are potential triggers of autoimmune diseases (ADs), especially in susceptible individuals. Mechanisms to establish molecular mimicry may include linear peptide homology, peptide modelling (i.e., three-dimensional structures), molecular docking analysis and affinity assessment of HLAs. Mechanisms based on structural vaccinology, immune mimicry and molecular mimicry are now available in combination with specific antigenic epitopes from proteomics in order to design targeted and highly specific pooled vaccines (89, 90). It is unavoidable that our studies of detailed mechanisms of molecular mimicry are limited by the long incubation period; epidemiological studies that have not yet established efficient linkages; the potential role of human genetics; limitations of animal models; and the limited technology available for the study of human T/B cell populations. We need to carry out more research on peptide dimensional analysis, which is becoming increasingly important for vaccine development and vaccination. This will facilitate a deeper understanding of the external effects that influence autoimmunity (91).

#### 5.2.2 Adjuvants

Adjuvants stimulate the immune system and thereby enhance the antigen-specific immune response induced by the vaccine, but do not have any specific antigenic properties of their own. Antigenic adjuvants in vaccines can enhance immunogenicity, thereby saving doses and strengthening the immune response. Adjuvant-induced autoimmune/inflammatory syndrome (Schofield’s syndrome, ASIA) was first described in 2011. Adjuvants are considered to be one of the pathogenic mechanisms of vaccine-associated autoimmune diseases, with stronger immune intervention in genetically susceptible individuals. Adjuvants include a variety of immunostimulatory factors (aluminium salts, medical implants, infectious agents, oil-in-water emulsions MF59, AS03 and AF03, etc.) (92). The To11-like receptor (TLRS) is a PRRS present on innate immune cells that recognises pathogens and initiates an immune response in the body. Adjuvants can bind to TLRs as TLR ligands and trigger innate and adaptive immune responses (93). Note that the adjuvant response is not the only way to trigger immunity. Currently, the number of relevant cases is small and the symptoms are variable. That is why we should establish a link from an epidemiological point of view. Vaccine histological studies and reduction of exogenous interference may improve the standard of adjuvant-associated diseases.

#### 5.2.3 Bystander activation

Vaccines stimulate innate immunity through non-specific pro-inflammatory antigens. At the same time, T or B cells can be activated non-specifically in an antigen-independent manner, i.e. bystander activation (94, 95). This process is independent of adjuvant-containing vaccines or adjuvant-only TCR connections. IFNI released during viral infection promotes clonal expansion and survival of CD8+ cells, which in turn favours the generation and maintenance of specific memory (96). It is characterised by a stimulatory response to lymphocytes independent of TCR/BCR specificity (97). The inflammatory environment, synergistic signalling ligands and reciprocal induced effects with neighbouring cells combine to promote bystander activation.

The results of a mouse model experiment showed that IL-2 and IL-7 were mediators of bystander activation in activated T cells *in vitro* (98). This conclusion was also verified by Boyman et al. (99). Di Genova et al. (100) found that a tetanus toxoid vaccine induced the activation of pre-existing bystander CD4+ Thmem cells. This is a response independent of tuberculin proteins or Candida albicans and does not occur in naïve CD4+ T cells. To further assess bystander activation of CD4+ T cells, van Aalst (101) et al. established a mouse model of TCR transgenic T cell transfer treated with different adjuvants, as well as performing splenectomy to sort and transfer specific CD4+ T cells into mice. This study revealed that transferred and labelled CD4+ T cells activated local bystanders and were unaffected by adjuvant.

These studies suggest that bystander activation can occur in an adjuvant-independent manner. In other words, there is currently no evidence that bystander activation necessarily requires an antigen-specific response to adjuvant. Currently, there is evidence that vaccination via bystander activation can lead to systemic lupus erythematosus (SLE) and rheumatoid arthritis (RA), among others. Therefore, we cannot rule out the possibility that COVID-19 vaccine may cause AIH through bystander activation; furthermore, more studies are needed to explore the mechanism of vaccine-mediated bystander activation on AIH.

#### 5.2.4 Epitope spread

Epitope (determinant cluster) spreading (ES) is an immune response to an endogenous epitope secondary to the release of self-antigen during a chronic autoimmune or inflammatory response. This endogenous epitope is distinct from the dominant epitope and does not cross-react. Epitope diffusion reflects the diversity of epitopes recognised by the immune system. It can be classified as intramolecular ES and intermolecular ES. Hu Z (102) et al. evaluated the circulating immune response in patients with stage IIIB/C or IVM1a/b melanoma after four years of postoperative treatment with NeoVax, an antigen-targeted long peptide vaccine (NCT01970358). They proposed that the vaccine could extend the range of specific cytotoxicity by inducing tumour cell killing through epitope spreading. As early as 1996, Vanderlugt CJ (103) and others have elaborated that epitope spreading plays an important role in autoimmune encephalomyelitis-associated CD4+ T cell-mediated multiple sclerosis mouse model. This suggests to us that epitope spreading is an important factor in the development of chronic pathogenesis of T cell-mediated and antibody-mediated autoimmune diseases. Currently, we know that autoimmune diseases such as systemic lupus erythematosus, multiple sclerosis, urticaria, and autoimmune blistering disease (104) are affected by epitope spreading. Further studies have shown that endocytic processing, antigen presentation, and somatic hypermutation are key molecular mechanisms that promote epitope spreading. Therefore, we should explore the broad characterisation of autoantibody targets to deepen our understanding of pathogenesis. The study of antigenic/epitope diversity is conducive to breakthroughs in vaccine-associated AIH.

Most of our known antibody epitope maps were developed using serum samples from late stages of the disease, which hinders the identification of key factors in the early stages of the disease. Autoantibodies to liver-kidney microsome-1 (LKM-1) due to type 2 autoimmune hepatitis react primarily with cytochrome P450 2D6 (CYP2D6).Hintermann E (105) et al. evaluated epitope spreading in a CYP2D6 mouse model and in AIH-2 patients, thus revealing the presence of immunodominant epitopes of homologous sequences in both in the early stages of the disease. Moreover, they demonstrated that epitope spreading begins at the immunodominant epitope and spreads to other sites. Therefore, this study suggests the importance of epitope spread in the pathogenesis of AIH.

#### 5.2.5 Polyclonal activation of B cells

The concept of non-specific polyclonal B cell activation as well as non-specific antibody production is well known. Acosta-Ampudia et al. found that autoantibody activity is associated with a proinflammatory state or autoimmunity (106). For example, acute infection with Mycoplasma pneumoniae is often accompanied by transient production of autoantibodies such as rheumatoid factor or cold agglutinins (107). Similarly, coronavirus infection triggers a B-cell cascade reaction leading to the production of similar autoantibodies. For instance, COVID-19-associated thrombotic events may be associated with an abnormal increase in antiphospholipid antibodies. Unfortunately, we do not yet fully understand the intensity of this immune response and the antibody effect. Therefore, we should not only consider the relationship between infection-induced autoantibodies and cytokine storms or inflammatory responses, which may be only a superficial and primary relationship. Rather, we should consider its fundamental nature, i.e., probing whether the B-cell cascade response is a hidden innate immune strategy to be triggered or an unpredictable mutation triggered by SARS-CoV-2 infection during viral infection. And, we should not ignore the extent to which autoantibody production influences the overall course of COVID-19. This can help us better understand the complexity of global pandemic epidemics and thus help us make decisions and early interventions.

Finally, mRNA vaccines offer the advantages of high potency, low cost, and high safety compared to traditional vaccines. Conversely, instability and inefficiency of *in vivo* delivery are technical challenges in mRNA vaccine development. For example, the mechanism of mRNA vaccine-induced AIH may include adjuvant and binding to pattern recognition receptor (PRR) (108). It has been shown that viral vector vaccines and inactivated vaccines can also generate molecular mimicry through different pathways, which in turn can exert unpredictable immune effects (109). Therefore, this technical challenge is yet to be overcome by more clinical trials and animal models.

### 5.3 Pharmacological liver injury or AIH?

Talotta et al. (110) suggested that women with pre-existing autoimmune diseases may be at risk of immune dysregulation when vaccinated with COVID-19. Akinosoglou K (111) et al. further elaborated on this issue, stating that COVID-19 vaccination does not trigger new immune-mediated adverse events, but that immune responses have the potential to trigger a genetically predisposed individual’s pre-existing existing underlying dysregulated pathways (112).

Nevertheless, most of the clinical evidence does not allow for a full spectrum diagnosis of AIH. Consequently, a hypothesis was born that AIH after COVID vaccination could be an acute drug-induced hepatitis with autoimmune properties or an AIH-like syndrome due to molecular mimicry of the vaccine, which is supported by comparisons with statin-induced liver injury (113). Recently Mann R (83) et al. reported a case of drug induced liver injury due to COVID-19 vaccine. A 61 year old female developed conjunctival jaundice, malaise, abdominal tenderness and fever 9 days after the second dose of Pfizer COVID-19 vaccine. Laboratory indices showed elevation of alkaline phosphatase and bilirubin, cholestatic phenotype and mild elevation of aminotransferases. Exceptionally, the patient was negative for all antibodies and liver biopsy showed only mild oedema and scattered infiltration of inflammatory cells. This is not consistent with the inference that the vaccine causes AIH. And the follow-up found that the patient’s symptoms and liver function indices gradually resolved. Of course, this study does not exclude the possibility of medical history as a risk factor.

Of course, the case reports are different and cannot be generalised. We can have multiple explanations for the post-vaccination liver lesions. For example, the association of viral spiking proteins with vaccine-induced spiking-specific receptors may lead to autoimmune dysregulation (1), vaccine-associated pharmacological liver injuries such as furotoxin or minocycline may also be characteristic of autoimmune hepatitis (114), and pregnancy, among others, is one of the risk factors for autoimmune disorders such as rheumatoid arthritis and thyroiditis (115–117).

Troublingly, although the mechanism of induction is not clear, the development of liver injury (VILI) after COVID-19 vaccination is similar to autoimmune hepatitis (AIH) in its clinical and histomorphological manifestations. However, surprisingly, recently Uzun S (118) et al. compared the two groups of samples by histomorphological assessment, whole and spatial transcriptome sequencing, multiplex immunofluorescence and immune complex sequencing. They finally found that TRBV6-1, TRBV5-1, TRBV7-6 and IgHV1-24 genes have different roles in the two. VILI had more pronounced centrilobular necrosis, increased activation of metabolic pathways, greater predominance of T- and B-cell clones, and more pronounced CD8+ T-cell infiltration compared with AIH, which had a predominance of CD4+ effector T-cells and CD79a+ B-cells and plasma cells. This study differs from previous conceptions by proposing that VILI may be a separate entity that is distinct from AIH and more closely related to drug-induced autoimmune hepatitis with a better prognosis.

So only vaccines based on minimal immune determinants specific to the pathogen and not present in the human proteome can ensure the safety and efficacy of the vaccine (52). Vaccine-induced AIH or transient drug-induced liver injury remains to be determined, but what is clear is that the association of SARS-CoV-2 vaccination with AIH is by no means coincidental. Certainly, the number and conditions of exposure events in the current study are not sufficient to form sufficient evidence to elaborate a causal relationship between COVID-19 vaccine and autoimmune hepatitis. It also suggests that we should be alert to and pay attention to the disease background, pathogenetic mechanisms, and coping strategies for the occurrence of AIH as a concomitant adverse event.

### 5.4 COVID-19 vaccination programme in AIH patients

Vaccination against SARS-CoV-2 significantly reduces the risk of COVID-19 death in AIH patients (119). Early reports have shown that immunosuppression, advanced chronic liver disease and liver transplantation lead to an increased risk of viral infection and impaired immune response after vaccination. A small prospective randomised controlled study (120) monitored antibody titres and spike-specific T-cell responses after SARS-CoV-2 vaccination in patients with AIH. They were found to have lower median antibody levels and 48% of antibody titres were below the 10% percentile of controls (9194 AU/mL, p<0.001). Also AIH patients demonstrated a higher risk of spike-specific T cell response failure and a lower frequency of spike-specific CD4+ T cells. The application of steroids (N=27, 7326 AU/mL, p=0.020) and MMF (N=14, 4542 AU/mL, p=0.004) can be considered as risk factors for them. Interestingly, all of the above phenomena were improved to varying degrees after booster vaccination. This suggests that our SARS -CoV-2 variant is more resistant to the neutralising effect of low titre antibodies and emphasises the need for routine assessment of antibody levels in AIH patients after vaccination and provision of booster vaccination according to individualised needs. On this basis, the appropriate adjustment of immunosuppressive agents may also be of significance for the improvement of vaccine response rates.

Barda N et al. (121), using a database from the largest healthcare provider in Israel, found that the third dose was more effective in preventing adverse outcomes of COVID-19 compared to patients who received only two doses of BNT162b2 mRNA vaccine at least 5 months earlier. In addition, vaccination with Pfizer or AstraZeneca vaccines can show 95% neutralisation of the Delta variant (122). Anti-Omicron antibody titres tended to rise significantly with the third dose of vaccine (123). Various such studies have demonstrated that the third dose of vaccine is effective in enhancing the post-vaccination immune response in immunosuppressed populations and may improve prognosis.

It is important to note that, despite the variety of vaccines, the antibody titres or activity required for effective resistance to infection are still not fully defined. Furthermore, immunosuppressive drugs such as steroids, mycophenolate mofetil or azathioprine suppress the strength of humoral or cellular immunity in the body to varying degrees. A similar phenomenon has been observed in patients with autoimmune rheumatic diseases (121), but the exact degree of immunity, such as the trend of cellular immunity, is not clear. As a result, it remains to be explored whether the adverse outcomes in autoimmune hepatitis patients infected with COVID-19 are due to the disease itself or to the immunological drugs. The standardised duration and efficacy of vaccine booster injections for the treatment of disease also remains to be analysed more comprehensively in larger prospective cohorts.

It is now known that comorbidities of liver transplantation also reduce the effectiveness of vaccination (124), and therefore revisions of their vaccination regimens are mostly advocated. Yet, to date, limited to sample size or deficiencies in control variables, there have been few breakthroughs in this direction as to the extent to which complications of AIH such as oesophageal varices, ascites and hepatic failure impact on neocoronary vaccination.

## 6 Mechanisms of genetic variation

The clinical progression of the disease is influenced by various factors, such as gender, age and genetics. In particular, the immune response to viral infections is strongly influenced by individual genetics. Nucleotide variants of single nucleotide polymorphisms (SNPs) (TNF-α, interferon (IFN)-λ, human leukocyte antigens (HLA), and interleukins) are involved in the innate and adaptive immune response, which is strongly associated with a causal link to the pathologic progression of AIH and COVID-19.

### 6.1 Cytokine SNPs

Research has indicated that the genotypes IL-6 rs1800795 CG+GG, rs1554606 (125), IL-8 rs2227306 CT+CC, and the C allele of IL-10 (rs1800896) (126) may serve as independent risk factors for severe progression and adverse outcomes in COVID-19 (127). Furthermore, the IL-6 rs1800795 GG genotype (128) and the IL-6R (rs12083537) GG genotype (129) can predict COVID-19 mortality. Conversely, compared to G/G-C/C, the IL-6 -174 G/C genotype may offer some protection against adverse COVID-19 progression (p = 0.03; OR = 0.41, 95% CI 0.18-0.96) (130). Likewise, IL-6 rs2069840 and rs2069837 (125) exhibit a similar protective effect (OR = 0.21, 95% CI = 0.07-0.69, *P*= 0.006) (131). Intriguingly, reports have shown that patients with autoimmune hepatitis (AIH) exhibit the IL-6 -174 GG genotype (132) and have significantly higher IL-6 polymorphism levels (133). Genetic testing has also demonstrated that patients carrying the IL-6 G (rs1800795), C (rs1800796), or G (rs1800797) alleles may be more susceptible to liver diseases (134). These findings suggest that SNPs in cytokine genes play a significant role in the pathological mechanisms underlying COVID-19-triggered AIH.

Additionally, the IL-1β(rs16944) CC genotype is significantly associated with severity and mortality in COVID-19, while IL-1βgene variation (rs1143634) may affect patient prognosis (135). In contrast, the IL1RN haplotype CTA and C/C variant of rs419598 can influence endogenous anti-inflammatory mechanisms, leading to attenuated cytokine release syndrome and reduced mortality in acutely infected males (136). Similarly, the rs1143634 SNP T allele exhibits a protective effect (130). Therefore, IL-1β rs16944 and IL-6R rs12083537 SNPs may serve as potential predictors of severity in SARS-CoV-2 infections.

Interestingly, a significant association exists between type 1 AIH and the rare 2308A allele (TNF*2), which is linked to increased TNF-α transcriptional activity (137). Other studies have shown that TNF-α -308G/A and -238G/A polymorphisms may contribute to increased susceptibility to autoimmune liver diseases (AILD) in Caucasians (128). Moreover, the A/A genotype of TNF-α (-308 G > A) positively correlates with TNF-α levels and susceptibility in AIH patients (138). Surprisingly, TNF-α (GG) significantly influences anti-SARS-CoV-2 spike protein IgG antibody levels (*P*= 0.005) (139). Individuals carrying the TNF-α G-308 A polymorphism A allele are more susceptible to COVID-19, and TNF-α (AA) is associated with a strong viral invasiveness (140). These findings align with SNPs in TNF-α in AIH. Furthermore, IFNs (125), particularly IFN-λ3 (IL-28B), are involved in antiviral functions. For instance, IFNλ4 rs73555604C can serve as a marker for COVID-19 severity (141). In SARS-CoV-2 infection, rs12979860 and rs1298275 were unfavourable genotypes in the context of hepatocellular carcinoma pathology, but further genetic information is needed.

Crucially, ACE2 polymorphisms can regulate immune responses and inflammatory processes. Hou Y et al. (142) examined DNA polymorphisms in ACE2 and TMPRSS2 (two key host factors for SARS-CoV-2) across approximately 81,000 human genomes and found that ACE2 polymorphisms are associated with cardiovascular and pulmonary diseases by altering angiotensinogen-ACE2 interactions. Meanwhile, p.Val160Met (rs12329760) in TMPRSS2 provides potential support for genetic susceptibility and risk factors in different individuals with COVID-19.

In summary, IL-6 rs1800795 and TNF-α G-308 A, as common SNPs in both diseases, may explain the genetic factors underlying COVID-19-induced AIH at the genomic level. However, no direct evidence currently links a specific ACE2 polymorphism to both AIH and COVID-19. Therefore, large-scale genomic sequencing can be actively conducted to determine if shared ACE2 polymorphisms exist between the two conditions. This may provide deeper insights into the causal associations between AIH and COVID-19 at the pathogenesis level, guiding the application of human genetic measures in combating virus-associated autoimmune diseases.

### 6.2 HLA polymorphisms

Specifically, there is a strong linkage disequilibrium between TNF*2 and HLA A1-B8-DR3 haplotypes, and an interdependence between TNF*2 and HLA DRB1*0301 (137). This reminds us that there are more genetic susceptibility mechanisms than just one SNP of cytokines. Studies on the nature of HLA gene polymorphisms may help to uncover the nature of disease associations. HLA polymorphisms, as a central component of the specific immune response, are involved in the recognition of foreign antigens, which triggers a precise immune response and plays a crucial role in warding off pathogen invasion and maintaining the health of the organism.

For instance, studies have demonstrated an association between HLA-A01 and both autoimmune hepatitis (AIH) and COVID-19 (143, 144), implying a notable immunogenetic similarity between these conditions. Nevertheless, disparities exist in the HLA polymorphic loci of AIH and COVID-19, with varying implications. Specifically, susceptibility to AIH type 1 (AIH-1) correlates with the presence of alleles such as DRB113, DRB10301, DRB30101, and DRB10401, among which DQB10301 exhibits protective effects (145). Conversely, AIH type 2 (AIH-2) is linked to HLA class II genes, particularly DRB107 and DRB103 (146), with the DQ locus emerging as a pivotal determinant in AIH-2 pathogenesis, notably DQB10201 as a primary susceptibility locus. Additionally, the HLA-DR (DRB107/DRB1*03) loci are implicated in diverse autoantibody expression profiles (131, 132).Utilizing Han-MHC reference panel analysis, Li Y et al. (147) revealed associations between AIH and HLA-B∗35:01, HLA-B∗08:01, and rs7765379 across the entire MHC region in the Han population, highlighting the significance of ethnic genetic specificity.

Notably, an HLA-B∗46:01 SNP has been implicated in COVID-19 susceptibility (125), while HLA-DQ rs7453920A and HLA-DPrs3077G have been suggested to influence COVID-19 severity (141).Furthermore, Bernasconi E et al. (148) conducted HLA testing on five patients who developed AIH following COVID-19 vaccination, all of whom harboured HLA DRB1 alleles (HLA DRB1*03, *11, *07, *13, *14) strongly associated with AIH susceptibility. These findings underscore the need to rigorously consider the hypothesis that COVID-19 infection or vaccination may precipitate AIH by modulating HLA polymorphisms, thereby presenting a variable and atypical clinical spectrum.

## 7 Mycophenolate salts applied to COVID-19 correlation AIH

The results of an open-label randomised controlled trial (149) demonstrated that in the treatment of autoimmune hepatitis (AIH), mycophenolate mofetil (MMF) and azathioprine achieved primary endpoints in 56.4% and 29.0% of cases, respectively (95% CI 4.0 to 46.7; *P*= 0.022). Notably, the biochemical remission rate at 24 weeks was significantly higher with MMF in combination with prednisolone for AIH treatment, and MMF exhibited better tolerability. Similarly, long-term prospective data (150) showed that compared to azathioprine, the MMF group had a lower 4-week non-response rate (*P*= 0.02), higher clinical benefit rates at 12 months and at the end of follow-up (86 vs. 71.8%; p < 0.05 and 96 vs. 87.2%; *P*= 0.03), fewer severe complications (3.8% vs. 18.8%; *P*= 0.0003), and greater eligibility for immunosuppression cessation (*P*< 0.05). Although MMF has demonstrated certain efficacy in the treatment of autoimmune diseases, the complex pathogenesis of COVID-19 and the fact that AIH is only one of its potential complications mean that the therapeutic effect of MMF in AIH caused by COVID-19 has not been widely validated. For AIH that emerges after COVID-19 vaccination, the therapeutic effect of MMF may vary among individuals.

Current case reports of COVID-19- and vaccination-induced AIH mostly utilize corticosteroids or ursodeoxycholic acid for treatment, with limited clinical application of immunosuppressants such as MMF or azathioprine. To date, multiple studies have indicated an inseparable relationship between COVID-19 and autoimmunity. Currently, immunosuppressive therapies for COVID-19 are at various stages of development, including corticosteroid trials, cytokine (IL-6/1) and tocilizumab (151, 152), granulocyte-macrophage colony-stimulating factor-targeted therapies (153), Janus kinase inhibitors (154), cell adsorbents, and combined antimalarial and antibiotic therapies (e.g., hydroxychloroquine and azithromycin) (155). There is an urgent need for clearer evidence to accurately assess the clinical value of immunosuppressants such as corticosteroids and MMF. Although corticosteroids are known to delay viral clearance without affecting mortality, and other therapies may highly depend on the timing of administration, their specific efficacy has not been proven.

It is noteworthy that MMF may decrease the immune efficacy of COVID-19-related vaccines. For instance, Tian Z et al. (156) found through multi-database analysis that AIH patients exhibited an attenuated immune response to COVID-19 vaccination, with mycophenolate mofetil reducing antibody responses. Likewise, Hartl J et al. (120) investigated the immune response of AIH patients to a third COVID-19 vaccination and found that median anti-SARS-CoV-2 antibody levels in AIH patients were significantly lower (10,908 vs. 25,000 AU/ml, *P*< 0.001), particularly in individuals receiving MMF (N = 14, 4542 AU/ml, *P*= 0.004) or corticosteroids (N = 27, 7326 AU/ml, *P*= 0.020). Additionally, liver transplant recipients have a lower ability to produce antibodies after anti-COVID-19 vaccination. Temporarily discontinuing immunosuppressive therapy such as MMF may be beneficial for humoral responses. Multivariate analysis (157) showed that temporarily suspending MMF for 2 weeks between the first and second doses of the Moderna mRNA-1273 vaccine could enhance antibody production during vaccination. The same principle, once validated, may be considered for use in formulating reference standards for other vaccination protocols in liver transplant patients. Therefore, the use of MMF before and after COVID-19 vaccination should be carefully considered. If MMF must be used, multiple studies support routine assessment of antibody levels in AIH patients to provide additional booster doses for those with inadequate responses.

## 8 Conclusion and perspective

In summary, the phenomenon of the association between AIH and COVID-19-related events in the context of the COVID-19 pandemic has generated widespread interest in the community. We found that having AIH was not associated with a higher risk of SARS-CoV-2 infection and poorer prognosis. Multiple lines of evidence suggest a triggering role for SARS-CoV-2 in autoimmune diseases, but we are still awaiting definitive elucidation of the relationship. In addition, the direction of SARS-CoV-2 mutation is unpredictable, and the lack of guidance from long-term large-sample trials has raised general concerns about the safety of the vaccine. We look forward to more in-depth studies on liver injury and AIH after vaccination to provide reliable guidance for clinical decision-making.

Improving disease progression and quality of life in patients with autoimmune disorders has become a critical issue to be addressed, provided that universal access to the importance of early diagnosis is achieved. Individualisation and personalisation of empirical antiviral regimens, vaccination regimens and appropriate adaptation of immunomodulatory therapies cannot be ignored. Although there are some under-incorporation of current vaccine drug trials, they are far superior to facing the significant risks that follow SARS-COV-2 infection. It is important to note that safe and effective vaccines should be administered and to raise awareness of the potential hepatic side effects of vaccines. This suggests that in-depth studies should be conducted to investigate the triggering mechanisms at the molecular biology level and the pharmacological toxicity of vaccines in the direction of autoimmunity. Information on biological targets expressed upstream and downstream of key genes may serve as a reference for specific immune markers, leading to the expansion and refinement of screening as well as the development of novel targeted drugs and their timing in combination. These may lead to breakthroughs in multi-case drug efficacy assessment and long-term prognostic monitoring to optimise overall disease management of autoimmune hepatitis in the post epidemic era.

We can conduct longitudinal surveys based on disease stage and follow-up capture, large-scale collection of disease data and analysis of visit trends and disease characteristics, and combined genetic analysis to create multi-ethnic biobanks. Initiatives such as studying the downstream mechanisms of specific autoantibodies may be beneficial in determining the clinical association of COVID-19 with AIH. The study of immune-targeted drugs in the therapeutic area of COVID-19-associated AIH may be an important development in the post epidemic era. Looking ahead, the phenomenon of liver immune disorders triggered by COVID-19-related events seems to offer us an extremely rare opportunity to explore more precisely how viral infections interact with autoimmunity.
