# Correction: Autoimmune hepatitis under the COVID-19 veil: an analysis of the nature of potential associations

**DOI:** 10.3389/fimmu.2026.1950438

**Published:** 2026-08-06

**Authors:** Chaojie Yu, Wenrui Wang, Qian Zhang, Zhenjing Jin

**Affiliations:** Department of Hepatopancreatobiliary Medicine, The Second Hospital of Jilin University, Changchun, Jilin, China

**Keywords:** SARS-CoV-2, COVID-19, autoimmune hepatitis, COVID-19 vaccine, immune-mediated liver injury

The funder “Natural Science Foundation of Jilin Province, (YDZJ202301ZYTS043)” to “Zhenjing Jin” was erroneously omitted. The **Funding** statement has been corrected to read:

“The author(s) declare that financial support was received for the research and publication of this article. Natural Science Foundation of Jilin Province (YDZJ202301ZYTS043) provided funding to author Zhenjing Jin.”

The original version of this article has been updated.

